# Deep Graph Clustering Framework Based on Confidence-Guided Graph Enhancement and Dual-Negative Sample Contrastive Learning

**DOI:** 10.3390/e28070763

**Published:** 2026-07-03

**Authors:** Qiuming Wang, Sheng Zhang, Bing Wu, Jiangnan Zhou, Chennan Wu, Yirong Zeng, Ka Sun, Chang Liu

**Affiliations:** School of Information Engineering, Nanchang Hangkong University, Nanchang 330063, China; 2404085404315@stu.nchu.edu.cn (Q.W.); 2404085410302@stu.nchu.edu.cn (B.W.); 2404085404312@stu.nchu.edu.cn (J.Z.); 2404085404323@stu.nchu.edu.cn (C.W.); 2504081200002@stu.nchu.edu.cn (Y.Z.); 70308@nchu.edu.cn (K.S.); lcsszz@nchu.edu.cn (C.L.)

**Keywords:** graph clustering, attributed graph, community structure, contrastive learning, data augmentation

## Abstract

Attributed graph clustering partitions nodes in an unsupervised manner by leveraging graph topology and node attributes. Existing deep methods face challenges including local structural bias, high noise in unsupervised graph editing, and insufficient discriminative ability for hard samples. To address these issues, we propose a deep graph clustering framework based on confidence-guided graph enhancement and dual-negative sample contrastive learning (CGEN). CGEN constructs a local–global dual-view representation learning module to fuse local neighborhood attributes with high-order global topological information. It then utilizes a confidence-guided conservative graph editing mechanism that integrates multiple constraints, specifically feature similarity, intra-cluster consistency, multi-view consistency, and pairwise node confidence, using a progressive update strategy for stable structural optimization. Furthermore, a dual-negative sample contrastive learning strategy dynamically adjusts the weights of attribute-confused and inter-cluster-confused negative samples to enhance discriminative ability near adjacent cluster boundaries. Extensive experiments on four benchmark datasets demonstrate that CGEN achieves highly competitive performance, outperforming the majority of state-of-the-art methods across core clustering metrics, thereby validating its effectiveness in addressing local structural bias, graph editing noise, and hard sample discriminative limitations.

## 1. Introduction

Graph-structured data naturally characterizes the relationships between entities in the real world. Due to its powerful representational capabilities, graph learning has become a crucial medium for analyzing complex systems, demonstrating significant research value and widespread applications in various downstream tasks [1]. As one of the core tasks in the field of graph learning, attributed graph clustering aims to group nodes into distinct partitions by leveraging both graph topology and node attributes in an unsupervised manner, thereby effectively revealing the latent community structures within the graph [2,3]. From a broader network science perspective, this objective is intrinsically linked to the classical community detection problem. Alternative graph-based methodologies in this broader domain have been widely explored, ranging from multiscale topological analysis that captures varying resolutions of community structures [4] to applied graph-theoretic approaches for complex real-world evaluations, such as group risk assessment in economic systems [5]. Building upon these foundational concepts, this task has widespread applications in social network analysis [1], recommendation systems [6], anomaly detection [7], and traffic data imputation [8]. Its core objective lies in learning low-dimensional node representations that possess both intra-cluster compactness and inter-cluster separability, as the representation quality directly determines the final clustering performance.

To enhance the node representation capabilities of attributed graph clustering, related studies have made significant progress. Early graph clustering approaches typically rely on traditional machine learning algorithms such as K-means [9] and spectral clustering [10], or optimize node representations by reconstructing the graph structure and node attributes [3,7,11]. However, these methods exhibit critical limitations: they rely on decoupled learning pipelines where representation extraction and clustering optimization remain isolated, and they struggle to adequately capture high-order topological information, leading to suboptimal clustering performance. The advent of graph convolutional networks (GCNs) [11] has provided a novel solution. By simultaneously leveraging node attributes and graph topology to propagate neighborhood information through layer-wise message passing, GCNs have emerged as the predominant methodological framework for deep graph clustering. With the successful development of contrastive learning in computer vision [12], graph contrastive learning has been introduced into attributed graph clustering tasks. Its optimization objective—pulling similar positive samples closer and pushing dissimilar negative samples apart—highly aligns with the core requirements of attributed graph clustering, progressively making it the dominant research direction. For instance, SCGC [13] constructs positive and negative sample pairs based on node adjacency relationships, while CCGC [14] utilizes pseudo-labels to treat nodes within the same cluster as positive samples and those in different clusters as negative samples, guiding representation learning toward a more clustering-friendly direction.

Despite the significant advancements achieved by recent methods, two key limitations remain, severely restricting further optimization of clustering performance. The first limitation is the local structural bias [15,16,17]. The vast majority of deep graph clustering methods rely on localized message-passing mechanisms (e.g., shallow GCNs) intrinsically driven by the homophily assumption, which presumes that connected nodes share similar attributes and cluster memberships. However, when real-world graphs exhibit heterophily—where edges frequently connect nodes from different underlying communities—or contain structural noise, this localized aggregation forces nodes to over-assimilate features from heterogeneous or anomalous neighbors, leading to ambiguous clustering boundaries and inter-cluster overlapping. Furthermore, intuitively stacking multiple GNN layers to capture higher-order global contexts is not a viable solution, as it inevitably triggers the well-documented over-smoothing problem, where the representations of all nodes exponentially converge to indistinguishable local minima. Consequently, a single local feature learning paradigm is theoretically trapped between vulnerability to local heterophily and the risk of global over-smoothing, failing to holistically characterize the cluster-level structural patterns across multi-hop paths. The second limitation lies in the instability of unsupervised graph enhancement and contrastive learning optimization, particularly when driven by unreliable pseudo-labels. On the one hand, existing structure optimization methods often directly reconstruct K-nearest neighbor graphs based solely on node similarity [18]. Updating the graph structure using this coarse-grained “similarity-means-edge” rule lacks constraints regarding cluster assignment credibility and multi-view consistency, which easily introduces spurious edges and generates structural noise. Furthermore, while some recent methods attempt to utilize clustering pseudo-labels to guide optimization, they largely overlook the severe challenges associated with pseudo-label noise. In the early iterations of unsupervised training, pseudo-labels are inevitably highly noisy and unstable. Directly relying on them without quality assessment leads to catastrophic error propagation: incorrect pseudo-labels will misguide graph editing to connect inter-cluster nodes and force the contrastive objective to falsely push away positive pairs (false negatives), ultimately deteriorating representation quality. On the other hand, mainstream contrastive learning methods predominantly adopt single structure-corrupted negative samples without distinguishing between hard and simple samples [13]. This results in the model’s insufficient discriminative ability against adjacent cluster confusion and attribute perturbations.

To address these core challenges, we propose a deep graph clustering method based on confidence-guided graph enhancement and dual-negative sample contrastive learning (CGEN). Our central research hypothesis is that combining conservative structural optimization with curriculum-based hard negative sampling creates a mutually reinforcing closed-loop. Guided by node confidence and multi-view consistency, this framework effectively mitigates graph noise and improves pseudo-label reliability, ultimately yielding high-quality representations with enhanced discriminative power for hard samples. Mechanistically, the proposed framework is expected to statistically improve clustering performance by first utilizing a local–global dual-view module to provide reliable structural priors. These priors strictly constrain the graph enhancement process to prevent the introduction of spurious edges. The resulting noise-suppressed topology subsequently serves as a robust foundation for dual-negative sample contrastive learning, which progressively pushes apart attribute-confused and inter-cluster-confused nodes to clarify ambiguous cluster boundaries and maximize overall inter-class separability. The main contributions of this work can be summarized as follows:

(1) We propose a local–global dual-view representation learning strategy. It combines a graph encoder for local feature extraction and a PPNP module for global topological diffusion, fusing their outputs to achieve both local discriminability and global stability. This strategy also provides multi-scale information for reliable pseudo-label generation and node confidence estimation;
(2) We design a confidence-guided conservative graph enhancement mechanism. It uses four constraints (similarity, intra-cluster consistency, multi-view consistency, and node confidence) to screen candidate edges and optimizes the graph structure via a progressive updating strategy. This mechanism effectively suppresses structural noise and aligns the graph with true cluster topologies;
(3) We propose a dual-negative sample contrastive learning approach. It constructs attribute-confused and cluster-prototype-based inter-cluster-confused hard negative samples and dynamically adjusts their weights during training. This approach alleviates early pseudo-label noise and significantly enhances the model’s discriminative ability for cluster boundaries and hard samples.

## 2. Related Works

### 2.1. Attributed Graph Clustering and Deep Multi-View Clustering

Attributed graph clustering has become a fundamental task in data mining due to its unsupervised nature and broad applicability. Graph clustering and community detection have a rich history rooted in network science, initially focusing on pure topological structures. Early graph partitioning approaches and spectral clustering techniques mapped nodes into low-dimensional spaces based on the eigenvectors of graph Laplacians [19]. Subsequently, modularity optimization principles drove the development of highly efficient heuristic algorithms, such as Louvain [20] and Leiden [21], which excel at identifying macro-level community structures in complex networks. While these foundational methods established the theoretical groundwork for graph mining, they predominantly rely on structural connectivity and struggle to naturally integrate rich, high-dimensional node attributes. To overcome this limitation and learn more expressive, non-linear representations, contemporary deep learning techniques were introduced to build upon these classical principles. By intrinsically fusing topological connectivity with node attributes, graph neural networks (GNNs) [22,23]—including graph auto-encoders (GAEs) [22], graph convolutional networks (GCNs) [24], and graph attention networks (GATs) [23]—have emerged as the predominant methodological frameworks. However, GCNs are highly susceptible to structural noise due to their over-reliance on unprocessed adjacency matrices. While GATs mitigate over-smoothing and preserve node distinctiveness via a learnable attention mechanism, their quadratic computational complexity limits scalability on large-scale graphs. To improve model efficiency, cross-modal distillation [16] and cross-encoding strategies [25,26] have been introduced to approximate the complex multi-path information fusion of heavier models using lightweight architectures.

In the realm of deep multi-view clustering, deep learning has significantly advanced the modeling of inter-view relationships. Early approaches like O2MAC [27] rely on a heuristic metric to select a single most informative view, which inadvertently discards complementary information embedded in other views. To achieve comprehensive view integration, subsequent models focus on view consensus: SGCMC [28] learns view consistency coefficients, while CMGEC [29] and DFP-GNN [30] fuse various adjacency matrices into a unified consensus graph. Despite these efforts, handling complex inter-view consistency and the training instability caused by attention mechanisms remain challenging, prompting the development of frameworks like SCMC [31] that explicitly balance view consistency and complementarity.

### 2.2. Graph Contrastive Learning and Data Augmentation

Graph contrastive learning (GCL) has revolutionized unsupervised graph representation by maximizing mutual information—pulling positive sample pairs closer while pushing negative pairs apart in the embedding space. Existing paradigms operate across various scales: GRACE [32] conducts node-level contrast using structure and attribute corruption, GraphCL [33] performs graph-level contrast via a readout function, and models like DGI [34] and GMI [35] maximize mutual information across multi-scale contexts. Recently, GCL4SR and AdaGCL [36,37] have integrated adaptive view generation and consensus mechanisms to mitigate data sparsity and model collapse.

A crucial prerequisite for effective GCL is data augmentation, which generates semantically distinct views. Traditional heuristic strategies (e.g., edge perturbation, random masking, and node-importance-based removal) primarily rely on random structural corruption. They lack task-specific optimization and often introduce uncontrollable noise without substantially enhancing cluster separability. Conversely, fully learnable augmentation schemes incur high computational costs, risk neglecting local node consistency by merely pursuing view differences, and struggle to weigh the contributions of augmented versus original views.

More critically, standard GCL suffers from the “false negative” problem, where nodes belonging to the same underlying category are erroneously treated as negative samples and pushed apart. Although recent methods attempt to refine sample construction using pseudo-labels—such as applying fully connected layers (SCAGC [38]), selecting k- nearest neighbors (CAGC [39]), or pseudo-label guidance (PGCL-DCL [40], MFCGC [41])—they remain limited in generating noise-free, reliable augmented graphs and widening the decision boundaries between hard classes. Addressing these specific bottlenecks in data augmentation and negative sampling is precisely the core motivation behind our proposed CGEN method.

## 3. Methods

### 3.1. Problem Definition

An undirected attributed graph can be denoted as G=(V,E,X), where $V=\{v_{1},v_{2},v_{3},\ldots ,v_{n}\}$ denotes the set of n nodes and $E=\{e_{1},e_{2},\ldots ,e_{M}\}$ denotes the set of M edges. The graph is characterized by two core matrices: the attribute matrix $X\in R^{n\times d}$ and the adjacency matrix $A\in R^{n\times n}$. Here, d is the dimension of node attributes, and each row $x_{i}\in R^{d}$ of X represents the attribute vector corresponding to node $v_{i}$. The adjacency matrix A is binary-valued: $A_{ij}=1$ if an edge connects nodes $v_{i}$ and $v_{j}$, and $A_{ij}=0$ otherwise.

Attributed graph clustering seeks to partition the graph nodes into k non-overlapping clusters $\left\{C_{1},C_{2},\ldots ,C_{k}\right\}$ by simultaneously exploiting topological structure and node features. The ideal clustering result ensures that nodes in the same cluster are densely connected and share similar attributes, while nodes in different clusters are sparsely linked and have distinct feature distributions.

### 3.2. Overall Framework

**Figure 1:**
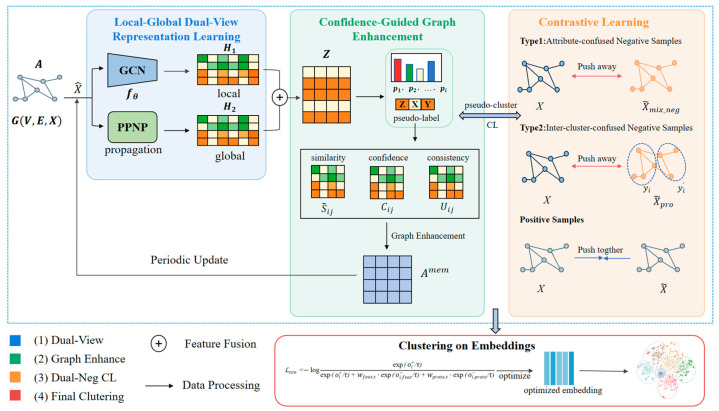
The overall framework of the proposed method.Points in different colors represent different clusters in the final clustering results.

As illustrated in Figure 1, the overall framework of the proposed attributed graph clustering method is as follows: First, we employ a graph encoder to extract attribute and structural features from the local neighborhood of each node based on the current graph topology. We then integrate the PPNP method to implement multi-step diffusion of high-order topological information for learning global node representations and obtain a comprehensive representation via feature concatenation of the two types of feature vectors. Subsequently, we construct pseudo-cluster states based on the comprehensive representations, and estimate the cluster assignment probabilities of nodes, node confidence scores, and multi-view consistency. Furthermore, we perform conservative graph enhancement on the original graph under the multiple constraints derived from these high-confidence cluster structural cues. Finally, we design contrastive optimization by constructing two types of negative samples to continuously improve the discriminative power of node representations for cluster structures. During the iterative training process, we gradually strengthen the consistency between the graph structure and the representation space, and perform clustering based on the optimal learned embeddings.

The graph enhancement process evolves from the coarse-grained “connect if similar” structural update paradigm to a conservative structural refinement strategy guided by “high confidence, consistency, and same-cluster membership”. Meanwhile, instead of relying on a single type of structure-corrupted negative samples to drive contrastive learning, we construct two complementary negative sample sets: attribute-corrupted negatives and inter-cluster confused negatives. We gradually increase the difficulty of hard negative samples in accordance with the training progress to mitigate the adverse impact of unstable early-stage pseudo-labels on model training. This design enables the model to gradually converge to a superior clustering representation space along a more stable training trajectory.

### 3.3. Local–Global Dual-View Representation Learning

To preserve both discriminative information of local neighborhoods and contextual information of high-order structures, we adopt a local–global dual-view representation learning strategy. First, we perform random feature augmentation on the input feature matrix X to obtain the augmented view $\tilde{X}=Aug(X)$. We then feed the augmented data into the encoder on the current graph structure to derive local node representations:

(1) $$H_{1}=f_{\theta }\left(\tilde{X},\hat{A}\right)$$

where we first add self-loops to the original adjacency matrix A to obtain $\tilde{A}=A+I$, and compute its corresponding degree matrix $\tilde{D}$, where ${\tilde{D}}_{ii}=\sum_{j}{\tilde{A}}_{ij}$. The normalized propagation graph is then defined as $\hat{A}={\tilde{D}}^{-1/2}\tilde{A}{\tilde{D}}^{-1/2}$, and $f_{\theta }(\cdot )$ denotes the parameterized graph neural network encoder.

The local representation captures the coupled attributes and structural relations between each node and its one-hop neighbors, which serves as the foundation for subsequent clustering representation learning. Nevertheless, pure local message propagation is constrained by its limited receptive field. As discussed in the introduction, directly addressing this by using alternative global mechanisms, such as deep convolutional stacking or unconstrained global attention, often drastically exacerbates over-smoothing and incurs prohibitive computational costs. To effectively capture latent structural relations across communities and multi-hop paths without losing node distinctiveness, we further adopt the predict-then-propagate (PPNP) [42] method based on the learned local representation. Unlike standard information diffusion models, PPNP is theoretically rooted in Personalized PageRank (PPR). The iterative propagation process is formulated as:

(2) $$Z_{m+1}=(1-\alpha )\hat{A}Z_{m}+\alpha H_{1}$$

where m is the propagation step and $Z_{0}=H_{1}$. Through this multi-step information diffusion, node representations aggregate high-order topological information to perceive macro-level cluster structures. Crucially, the teleportation probability α ensures a persistent structural connection to the original local root features $H_{1}$ during each step. This mathematical design fundamentally addresses the over-smoothing bottleneck: it guarantees that even with infinite-hop propagation (m→∞), the aggregated representations will not collapse into an indistinguishable state. Finally, the resulting global representation (denoted as $H_{2}$) is fused with $H_{1}$ to generate the comprehensive embedding Z. Compared to traditional multi-layer architectures, this elegantly bounded mechanism ensures that Z simultaneously preserves local node discriminability and global structural stability.

This design is more than a simple combination of two representations, and it provides multi-view information for the subsequent pseudo-cluster state modeling. Specifically, the comprehensive embedding Z characterizes the overall cluster structure, while $H_{1}$ and $H_{2}$ are used to evaluate clustering consistency from different structural perspectives. Benefiting from this multi-view collaboration mechanism, the subsequent confidence estimation is no longer limited to a single representation space and thus achieves more reliable node confidence evaluation across multiple structural scales.

### 3.4. Confidence-Guided Graph Enhancement

After obtaining the comprehensive embedding Z, we calculate soft cluster assignment probabilities to support subsequent graph enhancement and hard negative sample generation. We first perform K-means clustering on Z to obtain the set of cluster centroids $M=\{\mu_{1},\mu_{2},\ldots ,\mu_{K}\}.$ For each node $v_{i}$, we compute a distance vector $d_{i}=\{d_{i1},d_{i2},\ldots ,d_{iK}\}$ that measures its distance to all cluster centroids, and convert the vector into soft assignment probabilities. To eliminate the impact of inconsistent distance scales across different nodes, we conduct normalization as follows:

(3) $${\bar{d}}_{ik}=\frac{d_{ik}}{\sum_{j=1}^{K}d_{ij}}$$

The soft assignment probability is formulated as:

(4) $$p_{ik}=\frac{\exp(-{\overset{-}{d}}_{ik}/T)}{\sum_{j=1}^{K}\exp(-{\overset{-}{d}}_{ij}/T)}$$

where T>0 is the temperature parameter that controls the smoothness of the probability distribution. A smaller T produces a sharper distribution approaching hard assignment, while a larger T yields a more uniform distribution.

The preliminary pseudo-label of node $v_{i}$ is then obtained by $y_{i}=\arg\underset{k}{\max}p_{ik}$. Different from hard assignment strategies, this distance-to-probability mapping preserves the uncertainty of cluster membership. To quantify the reliability of pseudo-labels, let $p_{i,max}$ and $p_{i,sec}$ denote the largest and the second-largest cluster probabilities of node $v_{i}$, respectively. Drawing inspiration from margin-based sampling in active learning and confidence-aware pseudo-labeling [43], a robust confidence metric should account for both absolute certainty and decision boundary ambiguity. Therefore, we define the generalized single-view node confidence as:

(5) $$c_{i}=\omega p_{i,max}+(1-\omega )(p_{i,max}-p_{i,sec})$$

The first term, $p_{i,max}$, measures the absolute confidence of the model’s prediction. The second term, $\left(p_{i,max}-p_{i,sec}\right)$, acts as a margin penalty that explicitly penalizes nodes lying near the decision boundary between two overlapping clusters. A small margin indicates high ambiguity, even if the absolute probability is relatively high. By combining them, the metric effectively distinguishes nodes with ambiguous boundaries from those with definite cluster membership. The weighting parameter ω ∈ [0, 1] controls the relative importance of the two components. In our implementation, we assign equal importance to absolute confidence and boundary distinctiveness by setting ω=0.5, which is empirically validated to provide robust and optimal clustering performance (detailed sensitivity analysis is provided in Section 4.5). We next compute cluster assignments on the comprehensive view, local view, and global view, and refine node confidence via multi-view consistency. Let $y_{i}$, $y_{i}^{loc}$, and $y_{i}^{glo}$ represent the pseudo-labels derived from the comprehensive, local and global views, respectively. The consistency score is calculated by:

(6) $$s_{i}=\frac{1+I(y_{i}^{loc}=y_{i})+I(y_{i}^{glo}=y_{i})}{3}$$

where I(⋅) denotes the indicator function, which equals 1 if the internal condition holds and 0 otherwise. The final node confidence is defined as:

(7) $$c_{i}=\gamma \cdot \left(\frac{c_{i}^{cmb}+c_{i}^{loc}+c_{i}^{glo}}{3}\right)+(1-\gamma )\cdot s_{i}$$

where γ∈[0, 1] is a trade-off parameter balancing the probability-based confidence and the consistency-based reliable prior. In our experiments, we empirically set γ=0.5 (detailed sensitivity analysis is provided in Section 4.5). Nodes with consistent cluster assignments across multiple views will achieve higher confidence values. Compared with methods relying solely on single-view clustering results, the multi-view consistency constraint improves the quality of pseudo-labels and provides reliable structural priors for subsequent graph editing. We further define cluster representative points to construct high-quality hard negative samples. Suppose node $v_{i}$ belongs to cluster k, $d_{ik}$ is its distance to the corresponding centroid, and ${\bar{d}}_{k}$ denotes the average intra-cluster distance of cluster k. The node representativeness score is formulated as:

(8) $$r_{i}=c_{i}-\lambda \cdot \frac{d_{ik}}{{\bar{d}}_{k}+\epsilon }$$

where λ is a balance coefficient and $\epsilon =10^{-8}$ is a small constant to avoid division by zero. The representative node of cluster k is selected as:

(9) $$\;\rho_{k}=\arg\underset{\left\{i|y_{i}=k\right\}}{\max}r_{i}$$

This selection rule ensures that cluster representatives own high confidence and locate near the cluster center, which serves as valid references for constructing prototype negative samples. Graph structure optimization is a core component of our method. Unlike conventional approaches that reconstruct KNN graphs merely based on embedding similarity, we consider four criteria including similarity, intra-cluster affiliation, consistency and confidence to screen valid edges for graph enhancement. We first calculate similarities on the comprehensive view Z, local view $H_{1}$ and global view $H_{2}$, and average the results to obtain a unified similarity matrix:

(10) $$S=\frac{1}{3}\sum_{m=1}^{3}Z_{m}Z_{m}^{⊤}$$

where ${\tilde{Z}}_{m}$ denotes the normalized node representation of the m-th view. We remove diagonal elements to eliminate self-loops and apply a non-negative transformation to obtain the final similarity matrix:

(11) $$\tilde{S}=\phi \left(S-diag(S)\right)$$

in which ϕ(⋅) is the ReLU activation function. This scheme suppresses noise from a single view and enhances the stability of similarity estimation. In the candidate edge generation phase, we introduce intra-cluster constraints, pairwise node confidence and pairwise multi-view consistency. The intra-cluster mask is defined as:

(12) $$M_{ij}^{same}=I(y_{i}=y_{j})$$

The pairwise confidence and pairwise consistency are given by:

(13) $$C_{ij}=c_{i}c_{j},\;U_{ij}=s_{i}s_{j}$$

The candidate connection score is computed as:

(14) $$G_{ij}^{cand}={\tilde{S}}_{ij}\cdot M_{ij}^{same}\cdot U_{ij}\cdot I(C_{ij}\geq \tau_{add})$$

where $\tau_{add}$ is the confidence threshold. We only retain the top-$k_{i}$ connections that satisfy the neighbor number constraints to build the conservative candidate graph. An edge can be enhanced only when it meets all four requirements: high similarity, high consistency, identical cluster membership and high confidence, which is distinctly different from traditional similarity-only graph reconstruction. We adopt a conservative high-confidence enhancement strategy for graph updating. We strengthen reliable candidate edges while avoiding drastic destruction to the original graph. Let $A_{mem,t}$ denote the graph memory matrix at step t, and $G_{cand,t}$ denote the candidate edge matrix. The enhanced graph at current iteration is written as:

(15) $$A_{t}=Sym\left(\max\left(A_{mem,t},\eta_{add}G_{cand,t}\right)+I\right)$$

where $\eta_{add}$ is the edge weight coefficient, Sym(⋅) represents the symmetrization operation, and I is the identity matrix. To maintain continuous and stable structural updating, we update the graph memory via exponential moving average:

(16) $$A_{mem,t+1}=\tau A_{mem,t}+(1-\tau )A_{t}$$

where τ∈(0, 1) is the memory coefficient. Finally, we derive the normalized propagation graph:

(17) $$A_{mem,t+1}=D^{-\frac{1}{2}}A_{mem,t+1}D^{-\frac{1}{2}}$$

This mechanism enables gradual and stable structure optimization rather than one-shot drastic reconstruction, making it well suited for iterative learning in unsupervised graph clustering tasks.

### 3.5. Dual-Negative Sample Contrastive Learning

To further enhance the model’s discriminative ability for cluster structures, we employ two distinct types of negative samples in the contrastive learning stage, namely attribute-confused and inter-cluster-confused negative samples, rather than relying solely on a single negative sample type.

The attribute-confused negative samples are constructed via mixup between the original feature matrix $\tilde{X}$ and its perturbed version $P\tilde{X}$, where P is a random permutation matrix and the difficulty of negative samples is controlled by $\lambda_{t}\in [0,\;1]$:

(18) $${\tilde{X}}_{neg}=(1-\lambda_{t})\tilde{X}+\lambda_{t}P\tilde{X}$$

Inter-cluster-confused negative samples are designed to strengthen the model’s ability to distinguish boundaries between adjacent clusters. For any node $v_{i}$, let ${y_{i}}^{\prime }$ denote its alternative cluster and $\rho_{{y_{i}}^{\prime }}$ be the representative node of that cluster. The prototype negative sample is defined as:

(19) $$X_{i}^{pro-}=(1-\beta_{t})X_{i}+\beta_{t}X_{\rho_{{y_{i}}^{\prime }}}$$

where $\beta_{t}\in [0,\;1]$ is the mixing coefficient. ${y_{i}}^{\prime }$ denotes the second closest cluster to node $v_{i}$ based on the distance to cluster centroids. Unlike random negative samples, these prototype negatives are derived from cluster representatives that are “probabilistically suboptimal but semantically similar”, making them more challenging hard negative samples. They can more effectively push apart the representation boundaries between adjacent clusters.

Given the positive sample score $o_{i}^{+}$ of node $v_{i}$, the contrastive loss function is formulated as:

(20) $$L_{con}=-\log\frac{\exp(o_{i}^{+}/\tau )}{\exp(o_{i}^{+}/\tau )+w_{feat,t}\cdot \exp(o_{i,feat}^{-}/\tau )+w_{proto,t}\cdot \exp(o_{i,proto}^{-}/\tau )}$$

The dynamic weights $w_{feat,t}$ and $w_{proto,t}$ act as scaling factors for the negative terms in the InfoNCE denominator. In the early stage of training, $w_{feat,t}$ is large while $w_{proto,t}=0$, so the model focuses mainly on distinguishing attribute perturbations. In the later stage, $w_{proto,t}$ gradually increases to force the model to enlarge the distance from the prototypes of adjacent clusters.

### 3.6. Clustering

During training, the model first learns basic representations on a relatively stable graph structure and then refines the graph structure using well-trained embeddings. This design reduces the risk of distribution drift caused by frequent graph updates alongside representation learning. We adopt the contrastive loss as the optimization objective and continuously monitor the optimal model state during training. The optimized comprehensive node embedding is extracted under the optimal state, and clustering is conducted in this embedding space to produce the final results. Since graph editing and representation learning are jointly optimized in our method, the final clustering outcomes are not obtained by simply post-processing a single static representation. Instead, they are stable outputs yielded by iterative collaborative optimization between the representation space and graph structure.

### 3.7. Time Complexity Analysis

We comprehensively analyzed the computational complexity of the proposed CGEN framework, corresponding to the training procedure detailed in Algorithm 1. Let N be the number of nodes, |E| be the number of edges, d and h be the input and hidden dimensions, respectively, K be the number of clusters, and $K_{prop}$ be the PPNP propagation steps.

For Step 1 (local–global dual-view representation learning), the encoding requires $O(|E|dh+K_{prop}|E|h)$ per epoch. In Step 2 (joint optimization and graph editing), the pseudo-cluster state estimation via K-means takes O(I⋅NKh), where I is the number of iterations.

The most computationally intensive part is the graph enhancement module within Step 2. A naive dense pairwise similarity and consistency computation would strictly require $O(N^{2}h)$ time and $O(N^{2})$ memory, which severely hinders scalability on large graphs. To fundamentally address this limitation, as explicitly outlined in Algorithm 1, we design a cluster-aware sparse computing strategy in our practical implementation. Rather than computing global pairwise scores, we utilize the high-confidence intra-cluster masks to restrict candidate edge generation strictly within identically predicted clusters. Furthermore, we implement a sparse candidate capacity cap $M_{cap}$ (e.g., $M_{cap}\leq 256$) for each cluster based on node confidence descending order. This crucial engineering optimization bounds the graph editing complexity to $O(K\cdot M_{cap}^{2}\cdot h)$. Additionally, the graph memory update (via EMA) is only triggered periodically (every ΔT epochs), which significantly amortizes the structural updating cost across the entire training phase.

In Step 3 (curriculum dual-negative contrastive learning), the dual-negative contrastive optimization scales as $O(V_{neg}(|E|h+Nh))$, where $V_{neg}$ is the number of augmented views.

In summary, the computational complexity analysis reveals that a naive dense graph enhancement implementation would suffer from an intractable $O(N^{2}h)$ quadratic bottleneck. However, our proposed cluster-aware sparse computing strategy effectively resolves this issue by bounding the graph enhancement complexity to $O(K\cdot M_{cap}^{2}\cdot h)$, eliminating the quadratic dependency on node count. The overall average per-epoch time complexity of the optimized CGEN framework is $O\left(\left|E|dh+K_{prop}|E|h+I\cdot NKh+V_{neg}(|E|h+Nh)+\frac{1}{\Delta T}(K\cdot M_{cap}^{2}\cdot h\right)\right)$.
**Algorithm 1:** Training Procedure of **CGEN****Input:** Attributed Graph G=(V,E,X); Number of clusters K; Training epochs T; Pre-training epochs $T_{pre}$; Graph update interval ΔT; Hyperparameters (e.g., confidence threshold $\tau_{add}$, sparse capacity $M_{cap}$). **Output:** Final clustering result R.
1: Initialize: Model parameters θ$\;\mathrm{of}\;\mathrm{GCN}\;\mathrm{encoder},\;\mathrm{graph}\;\mathrm{memory}\;A_{mem}\leftarrow A$, and virtual prototype cache P←∅.2: 
for
 t=1 
to
 T do3:$\;\;\mathrm{Obtain}\;\mathrm{perturbed}\;\mathrm{features}\;\tilde{X}$ via random feature dropout;4:  **//Step 1: Local-Global Dual-View Representation Learning**5:$\;\;\mathrm{Compute}\;\mathrm{local}\;\mathrm{representation}\;H_{1}\leftarrow f_{\theta }(\tilde{X},A_{mem})$;6:$\;\;\mathrm{Compute}\;\mathrm{global}\;\mathrm{representation}\;H_{2}\leftarrow PPNP(H_{1},A_{mem})$ via multi-step propagation;7:$\;\;\mathrm{Obtain}\;\mathrm{comprehensive}\;\mathrm{representation}\;Z\leftarrow H_{1}+H_{2}$;8:  **//Step 2: Joint Optimization and Graph Editing**9:$\;\;\mathrm{If}\;t>T_{pre}$$\;\mathrm{and}\;\left(t-T_{pre}\right)\mathrm{mod}\Delta T=0$ **then**10:$\;\;\;\;\;\mathrm{Estimate}\;\mathrm{pseudo}-\mathrm{labels}\;Y,\;\mathrm{multi}-\mathrm{view}\;\mathrm{consistency}\;S\in R^{N}$$,\;\mathrm{and}\;\mathrm{node}\;\mathrm{confidence}\;C\in R^{N}$$\;\mathrm{using}\;Z,H_{1},H_{2}$;11:$\;\;\;\;\mathrm{Extract}\;\mathrm{cluster}\;\mathrm{representative}\;\mathrm{nodes}\;P_{curr}\leftarrow \{\rho_{k}{\}}_{k=1}^{K}$;12:$\;\;\;\;\;\mathrm{Construct}\;\mathrm{candidate}\;\mathrm{graph}\;G_{cand}$$\;\mathrm{via}\;\mathrm{cluster}-\mathrm{aware}\;\mathrm{sparse}\;\mathrm{computation}\;(\mathrm{bounded}\;\mathrm{by}\;M_{cap}$) under quadruple constraints: similarity, Y, S$,\;\mathrm{and}\;C\geq \tau_{add}$;13:$\;\;\;\;\mathrm{Update}\;\mathrm{graph}\;\mathrm{memory}\;\mathrm{via}\;\mathrm{EMA}:\;A_{mem}\leftarrow \tau A_{mem}+(1-\tau )G_{cand}$;14:    Update virtual prototype cache P$\;\mathrm{with}\;P_{curr}$;15:    **end if**16:    **//Step 3: Curriculum Dual-Negative Contrastive Learning**17:$\;\mathrm{Construct}\;\mathrm{attribute}-\mathrm{confused}\;\mathrm{negatives}\;{\tilde{X}}_{neg}$$\;\mathrm{and}\;\mathrm{inter}-\mathrm{cluster}\;\mathrm{prototype}\;\mathrm{negatives}\;X_{neg}^{pro-}$ using P;18:$\;\mathrm{Compute}\;\mathrm{InfoNCE}\;\mathrm{contrastive}\;\mathrm{loss}\;L_{con}$$\;\mathrm{using}\;\mathrm{dynamic}\;\mathrm{curriculum}\;\mathrm{weights}\;w_{feat,t}$$\;\mathrm{and}\;w_{proto,t}$;19:$\;\mathrm{Compute}\;\mathrm{cluster}\;\mathrm{guidance}\;\mathrm{loss}\;L_{clu}$ (KL-divergence) if enabled;20:    **//Step 4: Parameter Update**21:    Update encoder parameters θ$\;\mathrm{by}\;\mathrm{minimizing}\;L_{total}=L_{con}+L_{clu}$;22:**end for**23:Perform K$-\mathrm{means}\;\mathrm{clustering}\;\mathrm{on}\;\mathrm{the}\;\mathrm{optimized}\;\mathrm{embedding}\;Z^{*}$ to obtain R

## 4. Experiments

### 4.1. Datasets and Metrics

To validate the effectiveness of the proposed CGEN method, we conduct extensive experiments on four benchmark datasets, including Amazon Photo (abbreviated as AMAP [26]), CORA [7], CITESEER [15], and ACM [44] datasets. Among them, the AMAP dataset contains a large number of edges and can be regarded as a dense graph. The detailed statistics of the datasets are summarized in Table 1.

Four widely adopted clustering evaluation metrics are used to quantitatively assess the performance of all compared methods, and their mathematical definitions are provided below.

The accuracy (ACC) measures the overall proportion of correctly clustered samples:

(21) $$ACC=\frac{TP+TN}{TP+TN+FP+FN}$$

where TP (True Positive) and TN (True Negative) denote the number of samples correctly assigned to their respective classes, FP (False Positive) refers to samples incorrectly classified as positive, and FN (False Negative) represents samples misclassified as negative.

The normalized mutual information (NMI) quantifies the statistical dependence between ground-truth and predicted clusterings:

(22) $$NMI(U,V)=\frac{2\times I(U,V)}{H(U)+H(V)}$$

Here, I(U,V) measures the mutual information between the ground-truth cluster assignment U and the predicted cluster assignment V, while H(U) and H(V) correspond to the Shannon entropies of U and V, respectively.

The adjusted Rand Index (ARI) corrects the Rand Index for chance agreement:

(23) $$ARI=\frac{RI-E(RI)}{Max(RI)-E(RI)}$$

where RI denotes the original Rand Index that counts the fraction of pairwise sample agreements, E(RI) is the expected value of RI under random cluster assignments, and Max(RI) represents the maximum achievable value of RI for the given dataset.

The F1-score is the harmonic mean of precision and recall, which provides a balanced measure of clustering performance. The corresponding formulas are:

(24) $$F1=\frac{2\times Precision\times Recall}{Precision+Recall},\;Precision=\frac{TP}{TP+FP},\;Recall=\frac{TP}{TP+FN}$$

### 4.2. Experimental Setting

The proposed CGEN method is implemented using the PyTorch deep learning framework (version 2.0.0, Python 3.11). The local view encoder in the local–global dual-view module is a single-layer Graph Convolutional Network (GCN) with PReLU as the non-linear activation function. The model is trained end-to-end using the Adam optimizer. To stabilize training and mitigate the adverse effects of unstable early pseudo-labels, the training process is divided into two phases: a pre-training phase and a joint optimization phase. During the pre-training phase (first $T_{pre}$ epochs), the model initializes network weights using only the basic contrastive loss. In the joint optimization phase, pseudo-cluster state re-estimation and confidence-guided graph enhancement are performed every ΔT epochs.

Key hyperparameters are carefully tuned to optimize clustering performance across different datasets. Basic optimization parameters include the initial learning rate η, hidden layer dimension $d_{h}$, and weight decay coefficient. The smoothness of soft cluster assignment probabilities is controlled by the temperature parameter T. The confidence threshold for candidate edge addition is $\tau_{add}$, and the stability of graph structure updates is regulated by the exponential moving average memory coefficient τ. The upper bounds of dynamic scaling weights for attribute-confused and inter-cluster-confused negative samples are set to $w_{feat}$ and $w_{proto}$, respectively, which increase linearly with training progress to gradually approach the optimal clustering representation space. The optimal hyperparameter settings for each dataset are summarized in Table 2.

**Figure 2:**
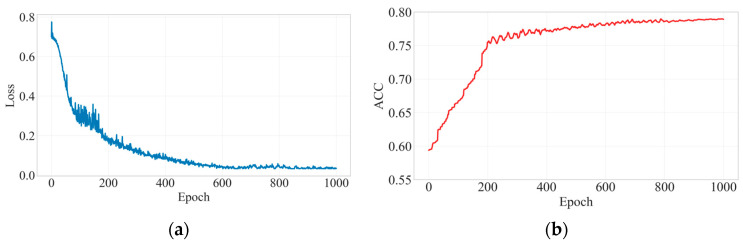
Convergence analysis of the proposed method on the CORA dataset across training epochs. (**a**) The variation in the training loss; (**b**) the variation in the clustering accuracy.

To verify the convergence of CGEN, we analyze the loss and accuracy curves on the CORA dataset (Figure 2). The loss decreases rapidly and then converges slowly, while the accuracy improves significantly within the first 200 epochs and then stabilizes. This indicates that the model can effectively extract structural features from graphs with structural noise without excessive training. Moreover, the accuracy continues to improve slightly as training progresses, confirming the effectiveness of the proposed graph enhancement module. To balance convergence and training efficiency, we set different training epochs for each dataset based on their size and structural characteristics: 400 epochs for ACM and CITESEER, 600 epochs for CORA, and 300 epochs for AMAP.

### 4.3. Performance Comparisons

In this subsection, we conduct experiments on four benchmark datasets and compare the performance with twelve baseline methods to validate the effectiveness of the proposed CGEN method. Specifically, the compared methods are categorized into two groups: classical graph clustering methods (K-means [9], GAE [22], DAEGC [2], AGE [3], SDCN [44]) and contrastive learning-based graph enhancement clustering methods (SCAGC [38], SCGC [13], CCGC [14], GDCL [45], ProDCL [46], HSAN [15], CDGC [47]). Notably, GDCL, ProDCL, and HSAN belong to hard sample contrastive learning approaches.

In Table 3, the best results are highlighted in bold, and suboptimal results are indicated by an underline. The data demonstrate that CGEN exhibits highly competitive performance, outperforming the majority of baseline models across all core evaluation metrics. From the macro perspective of the table, several key observations can be drawn: the clustering performance of contrastive learning (CL)-based methods comprehensively outperforms classical methods such as K-means and GAE, which fully verifies the effectiveness of self-supervised contrastive signals in excavating the latent semantic information embedded in graph structures and node attributes. Meanwhile, compared to standard CL methods, hard-sample-mining (HSM) based approaches including HSAN, GDCL, and ProDCL demonstrate pronounced advantages on specific datasets by focusing on challenging boundary nodes that are prone to misclassification, yet their performance fluctuates significantly across different topological structures, which further highlights the necessity of organically combining adaptive graph enhancement with targeted hard sample mining to achieve stable and superior clustering performance across diverse real-world graph types.

Notably, our proposed CGEN achieves superior clustering results compared with recent state-of-the-art baselines. Detailed comparisons are as follows:

1. **CGEN vs. SCGC**: CGEN significantly outperforms the classical CL baseline SCGC on the CORA and ACM datasets. For instance, on the ACM dataset, CGEN achieves improvements of 3.21%, 7.97%, 6.48%, and 3.08% across the ACC, NMI, ARI, and F1 metrics, respectively. SCGC utilizes a neighborhood-oriented contrastive loss that heavily relies on the raw topology. By introducing a conservative graph enhancement mechanism, CGEN effectively purifies structural noise and supplements missing connections, yielding more clustering-friendly representations on graphs with sparse or noisy topology;
2. **CGEN vs. HSAN**: While both methods focus on hard sample discrimination, CGEN demonstrates substantial advantages on the ACM and CITESEER datasets. Compared with HSAN, which relies purely on semantic-level hard sample mining without structural modification, CGEN leverages dual-view representations to generate high-confidence cluster prototypes. This progressive optimization of both the graph structure and the contrastive boundaries effectively addresses the limitations of handling complex topologies using metric-learning alone;
3. **CGEN vs. CDGC**: Compared to the most recent graph-augmentation baseline CDGC, CGEN maintains a consistent lead across most metrics, particularly on CORA (+3.23% in ACC) and ACM. This highlights the importance of the curriculum-style dual-negative sampling strategy, which prevents the severe optimization instability that often occurs in conventional graph enhancement methods during early training stages.

Despite its overall superiority, a rigorous and honest characterization of the results reveals specific limitations of CGEN on certain datasets. First, on the AMAP dataset, CGEN achieves an ACC of 77.23%, which marginally trails SCGC (77.48%). This performance gap exposes an architectural limitation of CGEN when handling highly dense topologies. AMAP features an extremely dense graph structure (119,081 edges for 7650 nodes). In such dense networks, local GCN aggregation inherently captures massive and highly overlapping structural contexts. Consequently, CGEN’s noise-suppressing conservative graph enhancement with strict quadruple constraints becomes overly cautious on dense graphs, limiting its ability to uncover informative latent edges compared to SCGC’s comprehensive exploitation of the dense global topology. Second, on the CORA dataset, CGEN’s F1 score (73.38%) falls below that of HSAN (75.11%), and it similarly trails CDGC slightly on the CITESEER F1 metric. Since the F1-score is particularly sensitive to class imbalance and the preservation of minority classes, this indicates that while CGEN’s graph editing improves overall accuracy (ACC) by eliminating noisy edges, its strict multi-view consistency constraints may inadvertently prune sparse but critical connections belonging to minority classes. Methods like HSAN, which avoid altering the original sparse topology, exhibit better boundary preservation for minority categories in these specific cases.

Meanwhile, to further statistically compare CGEN and existing methods, statistical significance testing was conducted to analyze modest margins. Specifically, p-values were calculated on all metrics of the four datasets between CGEN and the best baseline methods, which are $8.5\times 10^{-5}$, 0.0062, and 0.0031 comparing SCGC, HSAN, and CDGC, respectively. The p-values show that the performance differences are statistically significant since all are below the 0.05 threshold. These findings further demonstrate significant improvements in CGEN over the existing methods.

### 4.4. Ablation Studies

To comprehensively evaluate the individual contributions and synergistic effects of the core components in CGEN, we conduct ablation studies on two representative datasets, CORA and ACM. The evaluation is structurally divided into two parts: evaluating the overarching core modules and dissecting the specific constraints within the graph enhancement mechanism.

#### 4.4.1. Evaluating Core Modules

Taking the full CGEN model as the baseline, we construct five variants by sequentially removing or degrading key functional modules:

- **w/o Global**: Removes the global view PPNP module, and only uses GCN to learn local node representations for pseudo-label estimation and contrastive learning;
- **w/o GE**: Removes the confidence-guided graph enhancement module, and keeps the original graph topology fixed throughout training without structural updates;
- **w/o Conf**: Degrades the graph enhancement constraint mechanism by removing confidence and multi-view consistency constraints, and only constructs a traditional KNN enhanced graph based on intra-cluster masks and feature similarity;
- **w/o Proto**: Removes inter-cluster-confused prototype negative samples, and only uses attribute mixup to construct a single type of negative sample for contrastive learning;
- **w/o Curri**: Abolishes the curriculum dynamic weight scheduling and introduces all negative samples with fixed weights during the entire training process.

**Figure 3:**
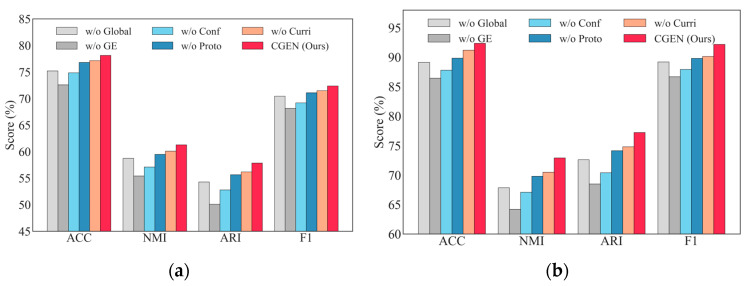
Ablation study results of the proposed CGEN method. (**a**) Performance comparison between the full model and its ablation variants on the CORA dataset; (**b**) performance comparison on the ACM dataset. All results are reported in terms of ACC, NMI, ARI and F1 scores.

The clustering results of these variants are presented in Figure 3. The full CGEN model achieves optimal performance across all metrics on two datasets. The removal or degradation of any component results in varying degrees of performance decline, indicating that all modules are mutually complementary and indispensable for the unified optimization framework.

Specifically, removing the graph enhancement module (w/o GE) incurs the most severe performance drop, with ACC and ARI decreasing by 4.92% and 7.74%, respectively, on the ACM dataset. This demonstrates that real-world attributed graphs inherently suffer from structural noise and sparse connections; representation learning alone is insufficient to break through the performance bottleneck. When degrading the strict enhancement constraints to a basic similarity-based graph construction (w/o Conf), the performance recovers slightly but remains significantly lower than the full model, accompanied by noticeable variance. This indicates that unconstrained, purely similarity-based edge addition easily introduces inter-cluster noisy connections, leading to structural drift. In contrast, our proposed quadruple constraints enable conservative and accurate topological updates, ensuring structural purity.

Regarding the dual-view architecture, the absence of the global view (w/o Global) reduces ARI by 3.55% on CORA, confirming that relying exclusively on local GCN message passing introduces structural bias and fails to capture multi-hop community structures. Furthermore, in the contrastive learning module, removing prototype hard negative samples (w/o Proto) reduces ACC by 1.52% on ACM. Unlike traditional mixup which merely improves robustness to attribute perturbations, our inter-cluster prototype negatives explicitly force the model to enlarge the distance between adjacent clusters, significantly enhancing inter-class discriminability. Finally, abolishing the curriculum weight scheduling (w/o Curri) reduces average performance and exacerbates training volatility. Since early-stage pseudo-labels are highly noisy, the premature introduction of hard negative samples disrupts the optimization direction. The curriculum mechanism ensures stable convergence by learning progressively from basic feature alignment to strict boundary discrimination.

#### 4.4.2. Dissecting the Graph Enhancement Module

While evaluating the core modules demonstrates the necessity of the graph enhancement module as a whole, it is crucial to rigorously isolate the contributions of its internal design elements. Therefore, we conducted a fine-grained ablation study specifically targeting the four constraints within the graph enhancement mechanism: feature similarity (w/o Sim), intra-cluster affiliation (w/o Intra), multi-view consistency (w/o Multi), and the node confidence threshold (w/o Thresh). The quantitative results are reported in Table 4.

As observed, removing any single constraint leads to a noticeable performance drop, confirming that all four components serve as essential topological filters. Notably, removing the intra-cluster constraint (w/o Intra) causes the most severe performance degradation (e.g., ACC drops by 3.58% on CORA and 3.73% on ACM). This indicates that without the strict boundary restriction of identical pseudo-clusters, the module inadvertently connects inter-cluster nodes, directly destroying the semantic separability of the graph. The w/o Thresh variant suffers the second-largest drop, highlighting the danger of allowing low-confidence nodes—which often reside near ambiguous decision boundaries—to introduce spurious structural noise. Finally, the absence of w/o Multi and w/o Sim also degrades performance, demonstrating that structural consensus across multiple views and underlying feature smoothness serve as indispensable auxiliary filters to guarantee the purity of the augmented topology.

### 4.5. Hyper-Parameter Analysis

To investigate the impact of core hyperparameters on the clustering performance of the proposed CGEN method, we conduct hyperparameter sensitivity experiments on the ACM dataset, focusing on the edge addition threshold $\tau_{add}$ in the graph enhancement module, as well as the weight of attribute-confused negative samples $w_{feat}$ and the weight of inter-cluster-confused prototype negative samples $w_{proto}$ in the dual-negative sample contrastive learning framework.

**Figure 4:**
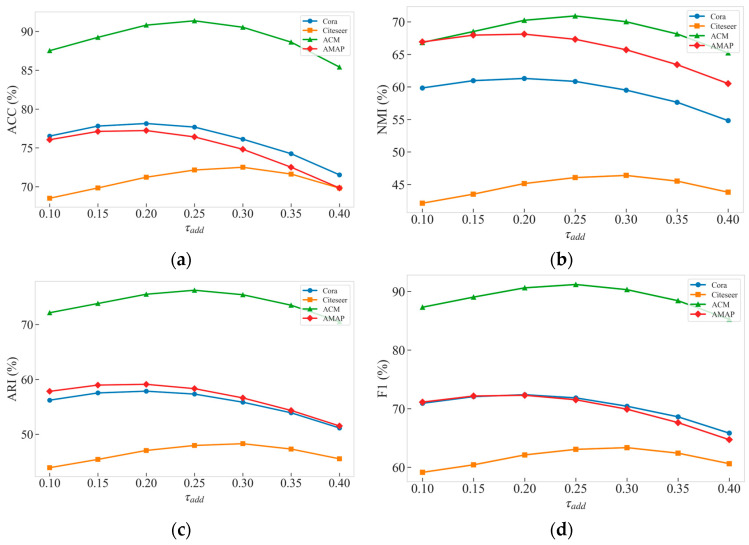
Hyper-parameter sensitivity analysis of the confidence threshold $\tau_{add}$ across four benchmark datasets (CORA, CITESEER, ACM, and AMAP). (**a**) The effect of $\tau_{add}$ on accuracy (ACC); (**b**) the effect of $\tau_{add}$ on normalized mutual information (NMI); (**c**) the effect of $\tau_{add}$ on adjusted Rand Index (ARI); (**d**) the effect of $\tau_{add}$ on F1 score.

The edge addition threshold $\tau_{add}$ controls the conservatism of graph structure optimization, and its performance variation is shown in Figure 4. When $\tau_{add}$ is set too small, a large number of low-confidence cross-cluster noisy edges are introduced into the graph structure, which easily triggers the over-smoothing problem caused by heterogeneous neighbors, resulting in poor ACC and ARI performance. As $\tau_{add}$ increases to the range of 0.2 to 0.3, the model achieves optimal performance. When $\tau_{add}$ exceeds 0.3, the stringent filtering conditions restrict information interaction among intra-cluster nodes, leading to fragmentation of the representation space and a significant degradation in clustering performance. These results demonstrate that an appropriate confidence threshold can effectively balance the connectivity and topological purity of the graph structure, verifying the rationality of the confidence-guided graph enhancement mechanism.

**Figure 5:**
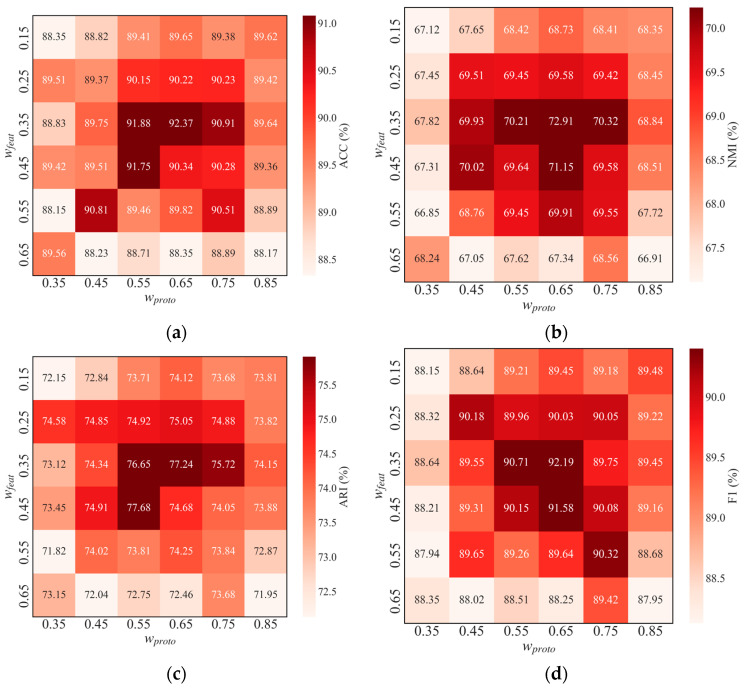
Hyperparameter sensitivity analysis of the dual-negative sample contrastive learning module on the ACM dataset. The heatmaps illustrate the clustering performance under different combinations of the attribute-confused negative sample weight $w_{feat}$ and the prototype negative sample weight $w_{proto}$. (**a**) Accuracy (ACC) results; (**b**) normalized mutual information (NMI) results; (**c**) adjusted Rand Index (ARI) results; (**d**) F1-score (F1) results.

To further analyze the synergistic optimization effect of the two types of negative samples, we plot a two-dimensional performance heatmap of $w_{feat}$ and $w_{proto}$, as shown in Figure 5. The experimental results indicate that using only a single type of negative sample cannot achieve optimal clustering performance. When $w_{feat}=0.35$ and $w_{proto}=0.65$, the model achieves an ACC of 91.3%, obtaining the global optimal performance. These findings fully validate the effectiveness of the curriculum-style dual-negative sample contrastive learning strategy. By reasonably allocating the weights of the two types of negative samples, the model simultaneously possesses the dual capabilities of resisting attribute perturbations and accurately delineating cluster structure boundaries, achieving comprehensive optimization of representation quality.

**Figure 6:**
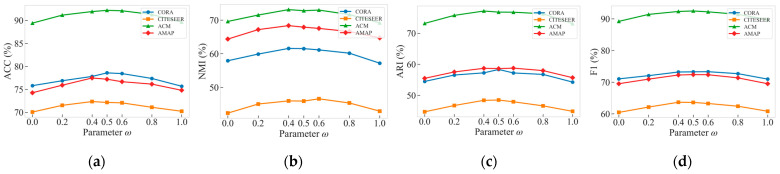
Hyperparameter sensitivity analysis of the confidence threshold ω on four benchmark datasets: CORA, CITESEER, ACM, and AMAP. (**a**) Accuracy (ACC); (**b**) normalized mutual information (NMI); (**c**) adjusted Rand Index (ARI); (**d**) F1-score.

**Figure 7:**
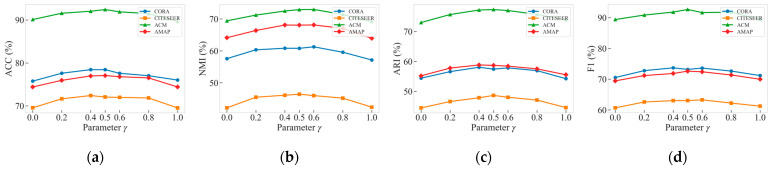
Hyperparameter sensitivity analysis of the confidence threshold γ on four benchmark datasets: CORA, CITESEER, ACM, and AMAP. (**a**) Accuracy (ACC); (**b**) normalized mutual information (NMI); (**c**) adjusted Rand Index (ARI); (**d**) F1-score.

We further evaluate the sensitivity of two core parameters governing the confidence calculation: the confidence weight ω (Equation (5)) and the multi-view consistency weight γ (Equation (7)), with results presented in Figure 6 and Figure 7, respectively.

The parameter ω balances the absolute prediction probability and the decision boundary margin. The framework achieves optimal and stable performance within the range of ω ∈ [0.4, 0.6]. When ω<0.4, an excessive emphasis on the boundary margin leads to overly conservative confidence estimation, resulting in insufficient topological enhancement. Conversely, ω>0.6 causes an over-reliance on absolute probabilities, failing to effectively penalize ambiguous boundary nodes and thereby introducing spurious edges into the augmented graph.

Similarly, the multi-view consistency weight γ exhibits a robust performance plateau between 0.3 and 0.6. Setting γ<0.3 unduly prioritizes cross-view structural consensus at the expense of probabilistic prediction reliability, while γ>0.6 diminishes the regularization effect of multi-view constraints, deteriorating pseudo-label quality. These findings demonstrate that both parameters possess a broad stability region. Our default setting of ω=0.5 and γ=0.5 optimally balances these complementary components, empirically validating the theoretical rationality of our parameter design.

### 4.6. Robustness to K-Means Initialization

As an unsupervised framework, the quality of early-stage pseudo-labels inevitably depends on the K-means initialization. To rigorously assess the robustness of CGEN under different initialization conditions, we compared the standard random initialization against the K-means strategy utilized in our default implementation, as reported in Table 5.

As observed, while random initialization introduces slightly higher variance due to suboptimal starting centers, CGEN still demonstrates remarkable robustness, maintaining highly competitive average scores without catastrophic collapse. This robustness is theoretically rooted in CGEN’s confidence-guided mechanism. Under poor initial label conditions, the model’s predictions exhibit high entropy (ambiguity). Consequently, the calculated node confidence scores ($c_{i}$) remain extremely low during the early epochs. Because our graph enhancement module strictly applies a confidence threshold ($\tau_{add}$), these early-stage noisy pseudo-labels are forcefully blocked from modifying the graph topology. The topological update is only triggered progressively as the self-supervised contrastive learning warms up and generates highly discriminative features. Furthermore, by adopting K-means as our default strategy, we maximize the initial distance between cluster centers, further accelerating stable convergence and minimizing variance across multiple random seeds.

### 4.7. Visualization Analysis

**Figure 8:**
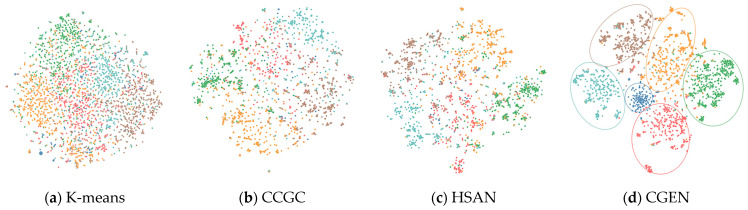
The visualization results of four methods on CITESEER dataset using t-SNE. Points in different colors represent different clusters in the clustering results.

To intuitively evaluate the quality of the learned node representations, we employ t-SNE visualization on the CITESEER dataset. For a comprehensive comparison, we select representative algorithms across different paradigms: a classical method (K-means), a contrastive learning (CL)-based method (CCGC), and a hard-sample mining method (HSAN). The corresponding visualization results are illustrated in Figure 8.

As observed, the traditional K-means method suffers from severe inter-class overlap, failing to effectively reveal the underlying community structure. Although recent deep clustering baselines, such as CCGC and HSAN, demonstrate improved aggregation capabilities, their representation spaces still exhibit noticeable inter-cluster overlap and blurred boundaries. In contrast, the proposed CGEN achieves a highly structured geometric distribution in the latent space.

The visualization clearly demonstrates that clusters generated by CGEN exhibit high intra-cluster compactness and well-defined decision boundaries, thereby maximizing inter-class separability. This improved spatial distribution confirms that CGEN effectively preserves the intrinsic structural relationships of the graph. Consequently, it enhances the discriminative power of the node representations and substantially mitigates the overlap between distinct semantic categories.

While t-SNE provides an intuitive 2D projection of the clustering distribution, it is inherently qualitative and can be sensitive to hyperparameter configurations (e.g., perplexity). To objectively quantify cluster compactness and separability in the original high-dimensional representation space, we further evaluate the learned embeddings using two standard internal clustering metrics: the Silhouette Coefficient (SC) and the Davies–Bouldin Index (DBI). A higher SC indicates that nodes are tightly matched to their own clusters and well-separated from neighboring clusters, whereas a lower DBI signifies higher intra-cluster compactness and superior inter-cluster separation.

As reported in Table 6, CGEN achieves the highest SC and the lowest DBI on the CITESEER dataset among the evaluated methods. Notably, compared to the hard-sample mining baseline HSAN, CGEN yields a significant improvement in both metrics. This quantitative evidence strongly corroborates our qualitative t-SNE observations, objectively validating that the proposed confidence-guided graph enhancement and dual-negative contrastive learning mechanisms effectively enlarge inter-class margins and tighten intra-class distributions within the high-dimensional latent space.

## 5. Conclusions

To address the core challenges in deep graph clustering, including local structural bias, interference from graph editing noise, and weak discriminative ability for hard samples, we propose a novel deep graph clustering method named CGEN, which integrates confidence-guided graph enhancement and dual-negative sample contrastive learning. Specifically, CGEN constructs dual-view representations by fusing local neighborhood features and high-order topological information, alleviating the model’s over-reliance on local structures. It designs a conservative graph editing strategy under four-fold constraints and optimizes the graph structure with a progressive update mechanism, effectively suppressing topological noise. Furthermore, a curriculum-style dual-negative sample contrastive learning module is built to enhance the discriminative ability for hard samples through dynamic weight adjustment. Finally, a co-training strategy with warm-up periodic updates is adopted to avoid distribution drift caused by pseudo-label instability, forming a positive closed loop between representation learning and structure optimization. Extensive experiments on CORA, CITESEER, ACM, and AMAP datasets demonstrate that CGEN exhibits highly competitive performance, outperforming the majority of state-of-the-art methods in terms of ACC, NMI, ARI, and F1 metrics, which verifies the effectiveness of the proposed method.

Despite the highly competitive performance demonstrated in this study, we acknowledge that our current experiments are constrained to four standard benchmark datasets. Consequently, the generalizability of the CGEN framework on ultra-large-scale graphs, heterophilous networks, dynamic graph structures, and complex real-world industrial datasets remains to be fully validated through broader experimental settings. Building upon the excellent clustering performance achieved by CGEN, our future work will focus on extending its applicability to more complex and dynamic real-world scenarios. On the one hand, we plan to integrate subgraph sampling, lightweight graph convolutions, and randomized algorithms to further enhance the model’s scalability and deployment efficiency in ultra-large-scale industrial networks. On the other hand, we aim to introduce non-parametric Bayesian models and density estimation techniques to enable the adaptive estimation of cluster numbers, thereby continually improving the model’s generalizability and flexibility in environments without prior structural knowledge.

## Figures and Tables

**Table 1:** Statistical summary of four datasets.

Dataset	Node	Edge	Class	Dimension
AMAP	7650	119,081	8	745
CORA	2708	5429	7	1433
CITESEER	3327	4732	6	3703
ACM	3025	13,128	3	1870

**Table 2:** Optimal hyperparameter settings for the proposed CGEN method across four benchmark datasets.

Dataset	η	$d_{h}$	T	$\tau_{add}$	τ	$w_{feat}$	$w_{proto}$
Cora	0.0010	512	0.30	0.22	0.92	0.35	0.65
Citeseer	0.0008	640	0.25	0.30	0.95	0.30	0.70
ACM	0.0008	512	0.30	0.24	0.90	0.35	0.65
AMAP	0.0003	384	0.45	0.18	0.88	0.45	0.89

**Table 3:** Comprehensive clustering performance comparison of state-of-the-art methods on CORA, CITESEER, ACM and AMAP datasets. Results are presented as ACC/NMI/ARI/F1, all are reported as mean ± standard deviation in percentage (%). Bold values indicate the best performance for each metric, and underlined values indicate the second-best performance. “---” indicates that the baseline methods did not provide experimental results on this dataset.

Metric	Method	CORA	CITESEER	ACM	AMAP
ACC	K-means	37.48 ± 2.71	54.41 ± 3.17	67.31 ± 0.71	27.22 ± 0.76
	GAE	64.96 ± 2.11	43.32 ± 0.80	84.52 ± 1.44	71.57 ± 2.48
	DAEGC	70.40 ± 0.36	67.20 ± 1.39	86.94 ± 2.83	75.96 ± 0.23
	AGE	71.62 ± 2.44	69.85 ± 0.45	90.63 ± 0.20	75.98 ± 0.68
	SDCN	35.60±2.83	65.96±0.31	90.45 ± 0.18	53.44 ± 0.81
	SCGC	73.88 ± 0.88	71.02 ± 0.77	89.16 ± 0.54	**77.48 ± 0.37**
	SCAGC	60.89 ± 1.21	61.16 ± 0.72	---	75.25 ± 0.10
	CCGC	73.88 ± 1.20	69.84 ± 0.94	90.01 ± 0.28	77.25 ± 0.41
	GDCL	70.83 ± 0.47	66.39 ± 0.65	---	43.75 ± 0.78
	ProDCL	57.13 ± 1.23	65.92 ± 0.80	---	51.53 ± 0.38
	HSAN	77.07 ± 1.56	71.15 ± 0.80	88.15 ± 0.51	77.02 ± 0.33
	CDGC	74.91 ± 1.78	70.12 ± 0.36	---	77.24 ± 0.87
	**CGEN**	**78.14 ± 0.41**	**72.50 ± 0.35**	**92.37 ± 0.67**	77.23 ± 0.65
NMI	K-means	24.60 ± 3.43	31.22 ± 3.22	32.44 ± 0.46	13.23 ± 1.33
	GAE	44.37 ± 2.97	21.70 ± 0.65	55.38 ± 1.92	62.13 ± 2.79
	DAEGC	52.80 ± 0.69	39.70 ± 0.93	56.18 ± 4.15	65.25 ± 0.24
	AGE	57.15 ± 0.91	44.62 ± 0.53	68.87 ± 0.49	65.38 ± 0.61
	SDCN	14.28 ± 1.91	38.71 ± 0.32	68.31 ± 0.25	44.85 ± 0.83
	SCGC	56.10 ± 0.72	45.25 ± 0.88	64.94 ± 1.44	67.67 ± 0.87
	SCAGC	39.72 ± 0.72	32.83 ± 1.19	---	67.18 ± 0.13
	CCGC	56.45 ± 1.04	44.33 ± 0.79	67.09 ± 0.53	67.44 ± 0.28
	GDCL	56.60 ± 0.36	39.52 ± 0.38	---	37.32 ± 0.28
	ProDCL	41.02 ± 1.34	39.59 ± 0.39	---	39.56 ± 0.39
	HSAN	59.21 ± 1.03	45.06 ± 0.74	63.73 ± 0.86	67.21 ± 0.33
	CDGC	58.16 ± 1.03	43.56 ± 0.35	---	67.12 ± 0.92
	**CGEN**	**61.30 ± 0.61**	**46.39 ± 0.64**	**72.91 ± 0.76**	**68.11** ± **0.67**
ARI	K-means	14.38 ± 1.95	28.54 ± 3.04	30.60 ± 0.69	5.50 ± 0.44
	GAE	39.41 ± 1.65	18.23 ± 1.18	59.46 ± 3.10	48.82 ± 4.57
	DAEGC	49.60 ± 0.43	41.00 ± 1.24	59.35 ± 3.89	58.12 ± 0.24
	AGE	48.23 ± 1.86	45.27 ± 0.61	74.38 ± 0.49	55.89 ± 1.34
	SDCN	7.78 ± 2.83	40.17 ± 0.43	73.91 ± 0.40	31.21 ± 1.23
	SCGC	51.79 ± 1.59	46.29 ± 1.13	70.76 ± 1.34	58.48 ± 0.72
	SCAGC	30.95 ± 1.42	31.17 ± 0.23	---	56.86 ± 0.23
	CCGC	52.51 ± 1.89	45.68 ± 1.80	72.81 ± 0.61	57.99 ± 0.66
	GDCL	48.05 ± 0.72	41.07 ± 0.96	---	21.57 ± 0.51
	ProDCL	30.71 ± 2.70	36.16 ± 1.11	---	34.18 ± 0.89
	HSAN	57.52 ± 2.70	47.05 ± 1.12	68.26 ± 1.24	58.01 ± 0.48
	CDGC	53.82 ± 2.25	44.85 ± 0.69	---	58.14 ± 0.82
	**CGEN**	**57.85 ± 0.76**	**48.28 ± 0.79**	**77.24 ± 1.67**	**59.10 ± 0.91**
F1	K-means	33.14 ± 4.46	41.20 ± 3.53	67.57 ± 0.74	23.96 ± 0.51
	GAE	63.60 ± 3.05	40.81 ± 0.82	84.65 ± 1.33	58.08 ± 1.76
	DAEGC	68.20 ± 0.57	63.60 ± 1.32	87.07 ± 2.79	69.87 ± 0.54
	AGE	68.06 ± 2.26	64.46 ± 0.37	90.61 ± 0.19	71.74 ± 0.93
	SDCN	24.37 ± 1.04	63.62 ± 0.24	90.42 ± 0.19	50.66 ± 1.49
	SCGC	70.81 ± 1.96	**64.80 ± 1.01**	89.11 ± 0.55	72.22 ± 0.97
	SCAGC	59.13 ± 1.85	56.82 ± 0.43	---	72.77 ± 0.16
	CCGC	70.98 ± 2.79	62.71 ± 2.06	89.98 ± 0.32	72.18 ± 0.57
	GDCL	52.88 ± 0.97	61.12 ± 0.70	---	38.37 ± 0.29
	ProDCL	45.68 ± 1.29	57.89 ± 1.98	---	31.97 ± 0.44
	HSAN	**75.11 ± 1.40**	63.01 ± 1.79	88.18 ± 0.50	72.03 ± 0.46
	CDGC	73.33 ± 1.86	**65.01 ± 0.39**	---	**73.02 ± 2.34**
	**CGEN**	73.38 ± 0.56	63.35 ± 0.95	**92.19± 0.58**	72.29 ± 0.52

**Table 4:** Results of the fine-grained ablation study designed to isolate the contributions of the four specific constraints in the graph enhancement module.

Dataset	Metric	w/o Sim	w/o Intra	w/o Multi	w/o Thresh	CGEN (Ours)
CORA	ACC	76.92	74.56	77.05	76.10	**78.14**
	NMI	59.85	56.40	60.12	58.74	**61.30**
	ARI	55.62	51.24	56.20	54.15	**57.85**
	F1	72.10	69.85	72.33	71.05	**73.38**
ACM	ACC	91.25	88.64	91.50	90.32	**92.37**
	NMI	71.18	67.50	71.65	69.80	**72.91**
	ARI	74.85	69.42	75.30	72.65	**77.24**
	F1	91.10	88.52	91.45	90.20	**92.19**

**Table 5:** Impact of K-means initialization strategies on clustering performance (Mean ± Std).

Dataset	Initialization	ACC	NMI	ARI	F1
CORA	Random Init	76.85 ± 1.12	59.92 ± 0.98	55.40 ± 1.25	72.15 ± 1.05
	K-means (Ours)	78.14 ± 0.41	61.30 ± 0.61	57.85 ± 0.76	73.38 ± 0.56
ACM	Random Init	91.05 ± 1.35	70.85 ± 1.22	75.10 ± 1.45	90.95 ± 1.18
	K-means (Ours)	92.37 ± 0.67	72.91 ± 0.76	77.24 ± 1.67	92.19 ± 0.58

**Table 6:** Quantitative evaluation of cluster separability on the CITESEER dataset using internal clustering metrics.

Method	SC	DBI
K-means	0.085	3.142
SCGC	0.142	2.531
HSAN	0.187	2.185
CGEN (Ours)	0.254	1.426

## Data Availability

All four benchmark datasets used in this study are publicly available. The processed data and implementation code are available on request from the corresponding author.

## References

[1] Zhou J., Cui G., Zhang Z., Yang C., Liu Z., Wang L., Li C., Sun M. (2020). Graph Neural Networks: A Review of Methods and Applications. AI Open.

[2] Wang C., Pan S., Hu R., Long G., Jiang J., Zhang C. Attributed Graph Clustering: A Deep Attentional Embedding Approach. Proceedings of the 28th International Joint Conference on Artificial Intelligence.

[3] Cui G., Zhou J., Yang C., Liu Z. (2020). Adaptive Graph Encoder for Attributed Graph Embedding. Proceedings of the 26th ACM SIGKDD International Conference on Knowledge Discovery & Data Mining (KDD ‘20).

[4] Liu Z., Barahona M. (2020). Graph-based data clustering via multiscale community detection. Appl. Netw. Sci..

[5] Ruzgas T., Stundziene A., Susniene R., Lukauskas M., Sinkevicius E., Staneviciute I., Zenceviciene J.A. (2025). Identification of the Company Groups in Assessing the Risk of Tax Evasion: A Graph Theory Approach. Comput. Econ..

[6] Ying R., He R., Chen K., Eksombatchai P., Hamilton W.L., Leskovec J. Graph Convolutional Neural Networks for Web-Scale Recommender Systems. Proceedings of the 24th ACM SIGKDD International Conference on Knowledge Discovery and Data Mining.

[7] Ding K., Li J., Bhanushali R., Liu H. Deep Anomaly Detection on Attributed Networks. Proceedings of the 2019 SIAM International Conference on Data Mining.

[8] Yu B., Yin H., Zhu Z. Spatio-Temporal Graph Convolutional Networks: A Deep Learning Framework for Traffic Forecasting. Proceedings of the 32nd AAAI Conference on Artificial Intelligence.

[9] Hartigan J.A., Wong M.A. (1979). Algorithm AS 136: A K-Means Clustering Algorithm. J. R. Stat. Soc. Ser. C Appl. Stat..

[10] Ng A.Y., Jordan M.I., Weiss Y. (2001). On spectral clustering: Analysis and an algorithm. Proceedings of the 15th International Conference on Neural Information Processing Systems: Natural and Synthetic, Vancouver, BC, Canada.

[11] Zhang X., Liu H., Li Q., Wu X.-M. Attributed graph clustering via adaptive graph convolution. Proceedings of the 28th International Joint Conference on Artificial Intelligence.

[12] He K., Fan H., Wu Y., Xie S., Girshick R. (2020). Momentum Contrast for Unsupervised Visual Representation Learning. Proceedings of the 2020 IEEE/CVF Conference on Computer Vision and Pattern Recognition (CVPR), Seattle, WA, USA.

[13] Liu Y., Yang X., Zhou S., Liu X., Wang S., Liang K., Tu W., Li L. (2024). Simple Contrastive Graph Clustering. IEEE Trans. Neural Netw. Learn. Syst..

[14] Yang X., Liu Y., Zhou S., Wang S., Tu W., Zheng Q., Liu X., Fang L., Zhu E. (2023). Cluster-Guided Contrastive Graph Clustering Network. Proc. AAAI Conf. Artif. Intell..

[15] Liu Y., Yang X., Zhou S., Liu X., Wang Z., Liang K., Tu W., Li L., Duan J., Chen C. (2023). Hard Sample Aware Network for Contrastive Deep Graph Clustering. Proc. AAAI Conf. Artif. Intell..

[16] Hu P., Chan K.C.C., He T. Deep graph clustering in social network. Proceedings of the 26th International Conference on World Wide Web Companion.

[17] Ren T., Zhang H., Wang Y., Ju W., Liu C., Meng F., Yi S., Luo X. (2025). MHGC: Multi-Scale Hard Sample Mining for Contrastive Deep Graph Clustering. Inf. Process. Manag..

[18] Lv P., Zhang C., Jia M., Liu Y. (2025). Deep Graph Clustering Method with Improved Cluster Structure Feature Learning. Appl. Intell..

[19] von Luxburg U. (2007). A Tutorial on Spectral Clustering. arXiv.

[20] Blondel V.D., Guillaume J.-L., Lambiotte R., Lefebvre E. (2008). Fast unfolding of communities in large networks. J. Stat. Mech. Theory Exp..

[21] Traag V.A., Waltman L., van Eck N.J. (2019). From Louvain to Leiden: Guaranteeing well-connected communities. Sci. Rep..

[22] Ding S., Wu B., Ding L., Xu X., Guo L., Liao H., Wu X. (2024). Towards Faster Deep Graph Clustering via Efficient Graph Auto-Encoder. ACM Trans. Knowl. Discov. Data.

[23] Salehi A., Davulcu H. (2020). Graph Attention Auto-Encoders. 2020 IEEE 32nd International Conference on Tools with Artificial Intelligence (ICTAI).

[24] Kipf T.N., Welling M. (2017). Semi-Supervised Classification with Graph Convolutional Networks. arXiv.

[25] Yang X., Tan C., Liu Y., Liang K., Wang S., Zhou S., Xia J., Li S.Z., Liu X., Zhu E. (2023). CONVERT: Contrastive Graph Clustering with Reliable Augmentation. Proceedings of the 31st ACM International Conference on Multimedia, Ottawa, ON, Canada.

[26] Liu H., Zhao W., Bao Z., Ye M., Shan C. (2025). Effective multi-view representation learning for single-view attributed graph clustering. Knowl.-Based Syst..

[27] Fan S., Wang X., Shi C., Lu E., Lin K., Wang B. (2020). One2Multi Graph Autoencoder for Multi-view Graph Clustering. Proceedings of The Web Conference 2020, Taipei, Taiwan.

[28] Xia W., Wang Q., Gao Q., Zhang X., Gao X. (2022). Self-Supervised Graph Convolutional Network for Multi-View Clustering. IEEE Trans. Multimed..

[29] Wang Y., Chang D., Fu Z., Zhao Y. (2023). Consistent Multiple Graph Embedding for Multi-View Clustering. IEEE Trans. Multimed..

[30] Xiao S., Du S., Chen Z., Zhang Y., Wang S. (2023). Dual Fusion-Propagation Graph Neural Network for Multi-View Clustering. IEEE Trans. Multimed..

[31] Fu L., Huang S., Zhang L., Yang J., Zheng Z., Zhang C., Chen C. (2024). Subspace-Contrastive Multi-View Clustering. ACM Trans. Knowl. Discov. Data.

[32] Zhu Y., Xu Y., Yu F., Liu Q., Wu S., Wang L. (2020). Deep Graph Contrastive Representation Learning. arXiv.

[33] You Y., Chen T., Sui Y., Chen T., Wang Z., Shen Y. (2020). Graph contrastive learning with augmentations. Proceedings of the 34th International Conference on Neural Information Processing Systems (NIPS ‘20).

[34] Veličković P., Fedus W., Hamilton W.L., Liò P., Bengio Y., Hjelm R.D. (2018). Deep Graph Infomax. arXiv.

[35] Peng Z., Huang W., Luo M., Zheng Q., Rong Y., Xu T., Huang J. (2020). Graph Representation Learning via Graphical Mutual Information Maximization. Proceedings of The Web Conference 2020 (WWW ‘20), Taipei, Taiwan.

[36] Zhang Y., Liu Y., Xu Y., Xiong H., Lei C., He W., Cui L., Miao C. Enhancing sequential recommendation with graph contrastive learning. Proceedings of the 31st International Joint Conference on Artificial Intelligence.

[37] Jiang Y., Huang C., Huang L. (2023). Adaptive graph contrastive learning for recommendation. Proceedings of the 29th ACM SIGKDD Conference on Knowledge Discovery and Data Mining.

[38] Xia W., Wang Q., Gao Q., Yang M., Gao X. (2023). Self-Consistent Contrastive Attributed Graph Clustering with Pseudo-Label Prompt. IEEE Trans. Multimed..

[39] Wang C., Pan S., Long G., Zhu X., Jiang J. (2017). MGAE: Marginalized graph autoencoder for graph clustering. Proceedings of the 2017 ACM on Conference on Information and Knowledge Management.

[40] Hu S., Zhang C., Zou G., Lou Z., Ye Y. (2025). Deep Multiview Clustering by Pseudo-Label Guided Contrastive Learning and Dual Correlation Learning. IEEE Trans. Neural Netw. Learn. Syst..

[41] Yang S., Liao Z., Chen R., Lai Y., Xu W. (2024). Multi-view fair-augmentation contrastive graph clustering with reliable pseudo-labels. Inf. Sci..

[42] Klicpera J., Bojchevski A., Günnemann S. Predict then propagate: Graph neural networks meet personalized pagerank. Proceedings of the 7th International Conference on Learning Representations.

[43] Rizve M.N., Duarte K., Rawat Y.S., Shah M. (2021). In Defense of Pseudo-Labeling: An Uncertainty-Aware Pseudo-label Selection Framework for Semi-Supervised Learning. arXiv.

[44] Bo D., Wang X., Shi C., Zhu M., Lu E., Cui P. (2020). Structural Deep Clustering Network. Proceedings of The Web Conference 2020, Taipei, Taiwan.

[45] Zhao H., Yang X., Wang Z., Yang E., Deng C. Graph debiased contrastive learning with joint representation clustering. Proceedings of the 30th International Joint Conference on Artificial Intelligence.

[46] Xia J., Wu L., Wang G., Chen J., Li S.Z. (2022). ProGCL: Rethinking Hard Negative Mining in Graph Contrastive Learning. arXiv.

[47] Yang X., Min E., Liang K., Liu Y., Wang S., Zhou S., Wu H., Liu X., Zhu E. (2024). GraphLearner: Graph Node Clustering with Fully Learnable Augmentation. Proceedings of the 32nd ACM International Conference on Multimedia, Melbourne, VIC, Australia.

